# Vitamin D and renal outcome: the fourth outcome of CKD-MBD? Oshima Award Address 2015

**DOI:** 10.1007/s10157-017-1517-3

**Published:** 2017-12-21

**Authors:** Takayuki Hamano

**Affiliations:** Department of Comprehensive Kidney Disease Research (CKDR), Osaka University Graduate School of Medicine, D11, 2-2 Yamadaoka, Suita, Osaka Japan

**Keywords:** Mineral and bone disorder, 25-Hydroxyvitamin D, Fibroblast growth factor 23, Proteinuria, Active vitamin D, Epithelial mesenchymal transition

## Abstract

Bone fracture, cardiovascular events, and mortality are three outcomes of chronic kidney disease-mineral and bone disorder (CKD-MBD), and the umbrella concept originally described for dialysis patients. The reported association of serum phosphorus or fibroblast growth factor 23 (FGF23) levels with renal outcome suggests that the fourth relevant outcome of CKD-MBD in predialysis patients is renal outcome. We found that proteinuria of 2+ or greater with a dipstick test was associated with low vitamin D status due to urinary loss of 25-hydroxyvitamin D (25D). Moreover, active vitamin D or its analogues decrease proteinuria. Given our finding that maxacalcitol does not repress renin, the reduction of proteinuria by this agent is likely due to direct upregulation of the nephrin and podocin in podocytes. Moreover, this agent downregulates the mesenchymal marker desmin in podocytes and blocks transforming growth factor—beta autoinduction, leading to attenuation of renal fibrosis in a unilateral ureteral obstructive (UUO) model. These facts are reminiscent of the suppression of epithelial–mesenchymal transition (EMT) by vitamin D. EMT blockage may explain our finding that vitamin D prescription in renal transplant recipients is associated with a lower incidence of cancer. We also reported that low vitamin D status and high FGF23 levels predict a worse renal outcome. However, administration of massive doses of 25D exacerbates renal fibrosis in UUO kidneys in 1alpha-hydroxylase knockout mice. Moreover, FGF23 inhibits 1alpha-hydroxylase in proximal tubules and monocytes. Taken together, local 1,25(OH)_2_D in the kidney tissue but not 25D seems to protect the kidney.

## Introduction

Chronic kidney disease (CKD)-mineral and bone disorder (MBD) is an umbrella concept originally described in dialysis patients. This syndrome is a systemic disorder composed of laboratory abnormalities, altered bone structure and bone fragility, and extra-skeletal calcification. It affects cardiovascular morbidity and mortality [1] and sometimes worsens quality of life of the affected patients due to bone fracture or bone pain. Because of increased levels of two phosphatonins including fibroblast growth factor 23 (FGF23) [2] and prevalent vascular calcification at the initiation of dialysis, the importance of CKD-MBD is currently recognized in the predialysis phase in patients with CKD. We and others have focused on key players in CKD-MBD such as 25-hydroxyvitamin D (25D) and the bone-derived hormone FGF23, because these are important determinants of levels of serum 1,25-dihydroxyvitamin D (1,25D), which is a crucial hormone that contributes to bone integrity [3] to prevent falls in patients with CKD [4].

## Cohort studies in CKD focusing on the vitamin D activation system

**Fig. 1:**
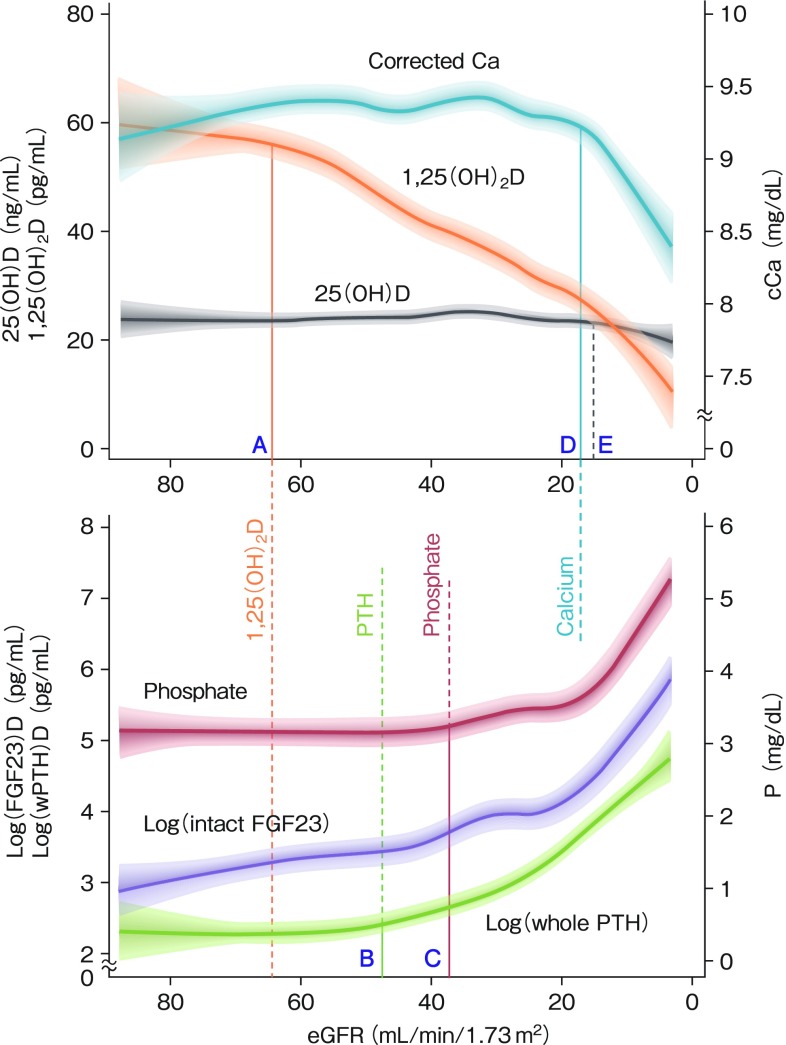
Cubic spline functions of the associations of eGFR with corrected calcium, 25D, 1,25D, log (intact FGF23), log (whole PTH), and phosphate. This is the summary of baseline data of OVIDS-CKD study [6]. The shadowed areas represent 95% confidence intervals for the fitted splines. The points A through E on the *x*-axis represent the thresholds of eGFR at which the slope of the curves (1,25D, log PTH, phosphate, corrected calcium, and 25D) departed from zero significantly. Above these thresholds, the slopes were not significantly different from zero. These thresholds were determined by computing the first derivatives of these curves. Serum 25D levels were constant across all eGFR categories. The slope of the log FGF23 curve is consistently positive from the earliest stage of CKD. As eGFR decreased, first serum FGF23 levels increased, followed by the decrease of 1,25D levels, and the increase in PTH and phosphate levels. Circulating 25D and corrected calcium levels were constant until eGFR < 20 ml/min per 1.73 m^2^

The first cohort study focusing on FGF23 was the Mild-to-Moderate Kidney Disease (MMKD) Study that enrolled 227 patients [5]. This is a study showing an independent relationship between plasma FGF23 levels and renal outcome [doubling of serum creatinine and/or end-stage renal failure (ESRD)]. However, this study enrolled only nondiabetic patients with CKD. Next, a cohort study called the Osaka Vitamin D Study in Patients with CKD (The OVIDS-CKD study) was conducted in Japan [6]. The study population consisted of 738 predialysis outpatients from nephrology departments including patients with diabetes. Because C-terminal fragments are circulating in serum from patients with ESRD [7], we measured intact FGF23 levels by a sandwich enzyme-linked immunosorbent assay system (Kainos Laboratories, Inc., Tokyo, Japan). This was in sharp contrast to the fact that C-terminal assays, mostly Immunotopics assay, were employed in most well-known cohort studies including the Chronic Renal Insufficiency (CRIC) study [8]. Because FGF23 levels as determined with the C-terminal assay are elevated by iron deficiency [Arrow (14) in Fig. 5] [9], which is often observed with erythropoiesis stimulating agent use, our study may have offered a more appropriate setting in which the association between the bioactive form of FGF23 and clinical outcomes can be explored. We examined the relationship between estimated GFR (eGFR) and levels of MBD markers including intact FGF23, whole parathyroid hormone (PTH), serum phosphate, calcium, 25D, and 1,25D using baseline data. Figure 1 shows the order of the change in these markers with eGFR decline. The earliest change in MBD markers was observed for intact FGF23, and the latest change was serum calcium levels. Circulating 25D levels were relatively constant across eGFR categories. In contrast, levels of 1,25D decreased linearly as renal function deteriorated, which is attributed to atrophy or dysfunction of proximal tubules and downregulation of 1alpha-hydroxylase activity by elevated FGF23. Whole PTH levels were stable between eGFR points A and B (Fig. 1) despite the decrease in serum 1,25D levels. This is compatible with the fact that FGF23 inhibits the secretion of PTH from parathyroid glands [10]. In our study, the rise in FGF23 preceded the rise in PTH levels. This order of changes in parameters is consistent with a previous animal study using rats treated with antibodies against glomerular basement membranes [11]. However, this sequence is currently reported to be dependent on vitamin D status [12]. In patients with vitamin D deficiency, PTH elevation precedes FGF23 elevation, whereas the opposite is true in vitamin D-repleted patients. The common finding is that the last marker to change regarding phosphate metabolism is serum phosphate. The explanation is that these compensatory increases in two phosphaturic hormones augment the fractional excretion of phosphate, leading to normalization of serum phosphate concentrations in early and moderate CKD. Clinical evidence for this explanation is seen in renal transplant recipients with persistent hyperparathyroidism. Serum phosphate levels are elevated by cinacalcet therapy, which reduces both PTH and FGF23 levels [13].

**Fig. 2:**
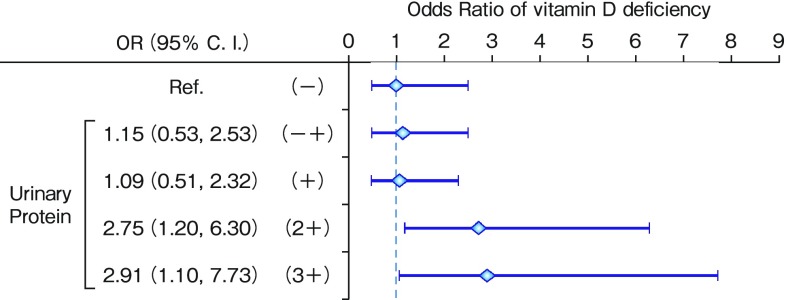
Significant associations of proteinuria with poor vitamin D status. Odds ratios for 25(OH)D insufficiency (defined as 25(OH)D < 30 ng/mL according to KDIGO guidelines) were calculated by logistic regression analysis. Urinary protein greater than 2+ by dipstick test remained significant even after adjustment for diabetes, sex, demographic factors (age, sex, and body mass index), laboratory data (Ca, P, and FGF23), medications (active vitamin D, glucocorticoid, and calcium carbonate), and seasonal diversion [14]

Next, we analyzed the risk factors for poor vitamin D status (defined as 25(OH)D < 23 ng/mL, median value in this cohort) with multivariate analysis. In addition to female sex and high PTH levels, moderate or high proteinuria (2 + or greater with a dipstick test) and diabetes were risk factors (Fig. 2) [14]. This is consistent with our basic science data showing that D-binding protein is lost with vitamin D in the puromycin aminonucleoside (PAN)-nephrosis rat model in which podocyte function is impaired [15]. Our data are also consistent with a clinical finding showing impaired megalin function in diabetic patients, even those with microalbuminuria [16], because megalin is indispensable for vitamin D reabsorption by proximal tubules.

## Vitamin D status and FGF23 levels as predictors of clinical outcomes

**Fig. 3:**
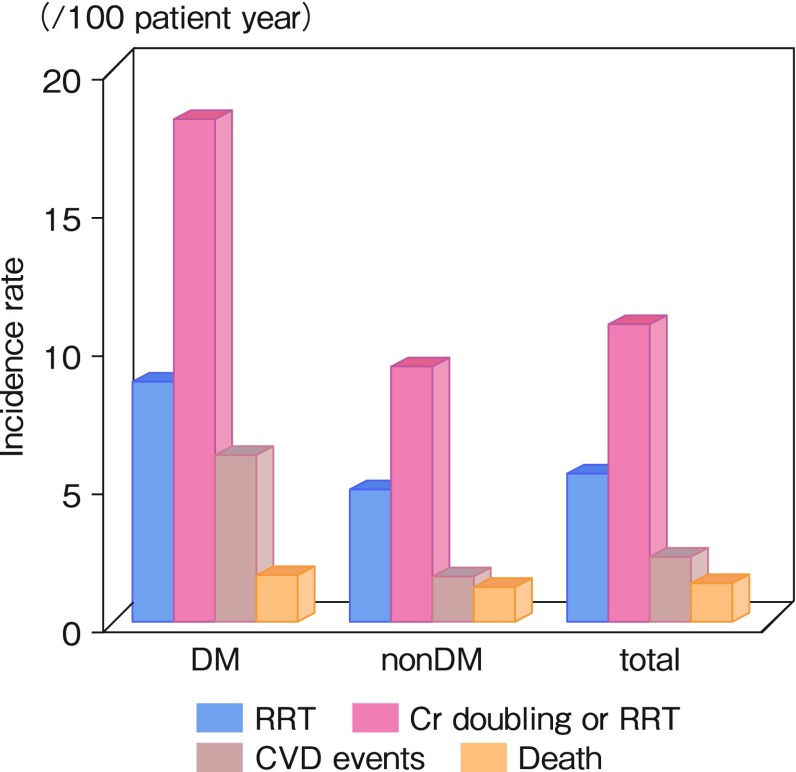
Incidence rate of each clinical outcome in Japanese patients with CKD stratified by diabetes. The incidence rate of renal replacement therapy (RRT), CVD events (stroke, coronary artery disease, congestive heart failure, aortic disease, and peripheral artery disease), and death stratified by diabetes is shown. The incidence of RRT was much higher than those of CVD events or death in Japanese patients with CKD [6]. Given this higher incidence and reported associations of vitamin D status and FGF levels with renal outcome, the fourth outcome of CKD-MBD for Japanese patients might be RRT

Low 25D levels may be an independent inverse predictor of CKD progression and death in predialysis Caucasian patients [17]. This study was limited, because a small number of renal outcome (< 50) led to very low statistical power, and neither PTH nor FGF23 was accounted for. Therefore, it is unclear which parameters (25D or PTH) predict renal outcome, resulting in ambiguous clinical implications. All six MBD-related parameters (calcium, phosphate, PTH, FGF23, 25D, and 1,25D) were thus measured simultaneously in the OVIDS-CKD study. With a median follow-up of 4.4 years (interquartile range 4.0–4.6 years), only 58 (7.9%) patients died. The number of patients achieving the renal endpoint was 213 (28.9%), of whom 156 reached a doubling of serum creatinine and 146 began renal replacement therapy (RRT). The incident rate of RRT, doubling of serum creatinine or RRT, cardiovascular disease (CVD) events, and death are shown in Fig. 3, stratified by the presence of diabetes. Here, CVD events contain stroke, coronary artery disease, congestive heart failure, aortic disease, and peripheral artery disease. Analogous to the findings in hemodialysis patients in the dialysis outcomes and practice patterns study (DOPPS study) [18], the incidences of CVD or death in Japanese patients with CKD were much lower than those in their western counterparts. A significantly higher number of events were observed for renal outcome, and we thus conducted multivariate analysis with renal events as a dependent variable to explore the independent association with MBD markers. The significant risk factors for renal outcome were high FGF23 levels and poor vitamin D status after extensive adjustment for the traditional factors such as proteinuria, anemia, and blood pressure. Of note is that these risk factors remained significant even after adjustment for serum phosphate and PTH and that serum 1,25D levels were not associated with CKD progression. The CRIC study reported that elevated FGF23 levels are independently associated with a significantly higher risk of ESRD only among patients with eGFR ≥ 30 [19]. In our study, increased FGF23 levels were significantly associated with CKD progression (doubling of serum creatinine or initiation of dialysis) among all subjects (not stratified by eGFR). In the MMKD study that enrolled only nondiabetic patients with mild-to-moderate kidney disease, both intact and C-terminal FGF23 levels independently predicted renal outcome (same definition as our study) [5]. In this study, the area under the curve appeared larger for the C-terminal assay than for intact FGF23. The reason may be that FGF23 levels measured with the C-terminal assay showed a significantly lower intra-individual variation than intact FGF23 levels [20]. In the OVIDS-CKD study, the patients were categorized into four groups by the median 25D and FGF23 levels (23.0 ng/mL and 49.5 pg/mL, respectively) to examine their combined effects. In a multivariable Cox model, the High FGF23–Low 25D group (hazard ratio 2.52; 95% confidence interval 1.13–5.62) was significantly associated with CKD progression compared with the Low FGF23–High 25D group as a reference.

Similar findings were observed in patients on maintenance hemodialysis with different clinical outcomes. The Hemo Study, which used time-dependent Cox regression models with repeated yearly measures, revealed that a low vitamin D status and high FGF23 levels are associated with infectious and cardiac deaths [21]. Again, similar to our study, no significant associations of 1,25D with clinical outcomes were observed in time-dependent or fixed-covariate Cox models. These observations can be explained by animal data showing that FGF23 impairs neutrophil recruitment [22]. Intriguingly, this basic research study showed that FGF23 neutralization during CKD in murine models restores leukocyte recruitment and host defense against *E. coli* infection. In addition, with surrogate outcomes, a similar relationship was observed in predialysis patients with CKD. In the CRIC study, increased left ventricular (LV) mass and cavity dilatation were observed in patients with low 25D and high FGF23 [23]. In participants with an FGF23 level higher than the median, each doubling of 25(OH)D was associated with a 2.5% lower LV mass. This association was less pronounced with FGF23 levels below the median (0.4%). Conversely, in participants with deficient 25D levels (< 20 ng/mL), each doubling of FGF23 was associated with a 3.4% greater LV mass compared with only a 1.6% difference in participants with sufficient 25D.

**Fig. 4:**
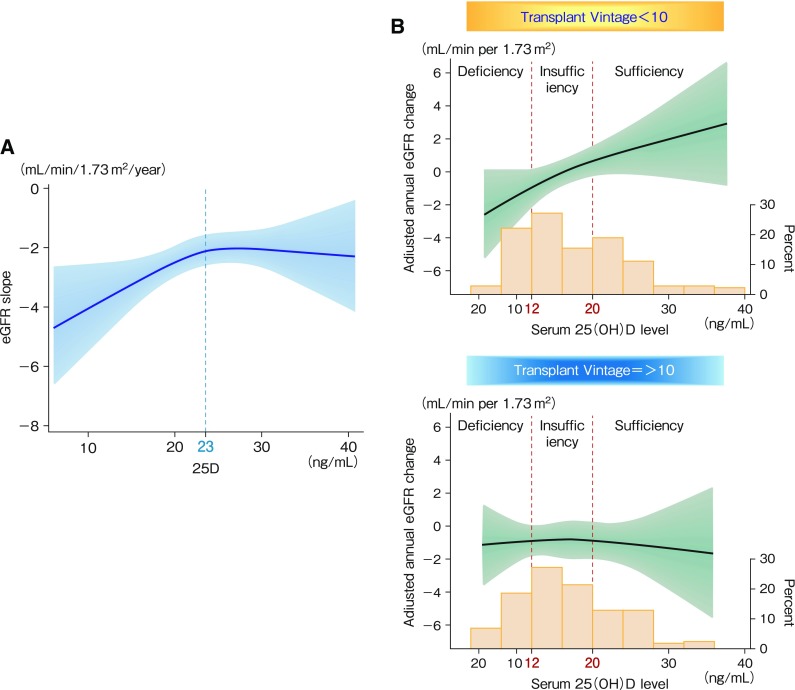
Association between vitamin D status and annual eGFR change in multivariate regression analyses in patients with CKD and renal transplant recipients. A non-linear relationship of serum 25D levels with adjusted eGFR slope (annual eGFR change) was observed in outpatients with CKD in a nephrology department [24] (**a**). Adjusted annual eGFR change and distribution of serum 25D concentration at less than 10 years and 10 or more years after transplantation are shown [25] (**b**). Robust linear regression with cubic spline functions was applied

Of note in our study, a non-linear relationship was observed between serum 25(OH)D levels and the annual eGFR slope after adjusting for confounders both in predialysis patients with CKD [24] and renal transplant recipients with transplantation vintage less than 10 years [25] (Fig. 4). This is also true of subjects with normal renal function in a community-based study [26].

## Vitamin D and epithelial–mesenchymal transition (EMT)

Active vitamin D or its analogues decrease proteinuria as confirmed by a recent meta-analysis [27]. Based on the VITAL study that showed that paricalcitol decreases proteinuria and systolic blood pressure [28], as well as some animal studies [29], many nephrologists attribute the effect of active vitamin D analogues to repression of renin. If so, administration of active vitamin D is not necessary to reduce proteinuria in the era of the direct renin inhibitor, aliskiren. However, given our finding that maxacalcitol does not repress renin [30], the reduction in proteinuria by this agent can be attributed to direct upregulation of nephrin and podocin [15], the slit diaphragm components in podocytes. This is also true for calcitriol, which acts on the vitamin D responsive element in the proximal nephrin promoter to stimulate nephrin expression in podocytes [31]. Paricalcitol also upregulates nephrin, podocin, and WT1 by inhibiting Wnt/β-catenin signaling [32]. Maxacalcitol also downregulates the mesenchymal marker desmin in podocytes [15]. These facts are reminiscent of the suppression of EMT by vitamin D analogues, since the reduced expression of nephrin and podocin can be regarded as the loss of the epithelial feature of podocytes. In fact, blockage of EMT by calcitriol and paricalcitol was confirmed by other researchers [33]. In addition, we confirmed that maxacalcitol represses Snail, the key transcription factor that regulates EMT. Moreover, in our study, maxacalcitol blocks transforming growth factor (TGF)-β autoinduction leading to attenuation of renal fibrosis in a unilateral ureteral obstructive (UUO) model [30] just like paricalcitol [34]. Regarding renal fibrosis, Ito et al. showed that 1,25D treatment prevents renal fibrosis by suppressing TGF-β-SMAD signal transduction. Moreover, they generated two synthetic ligands that selectively inhibit TGF-β-SMAD signal transduction without activating vitamin D receptor (VDR)-mediated transcription or causing hypercalcemia [35]. In other words, a non-classical VDR pathway suppresses renal fibrosis. These agents are very promising as therapeutic tools for CKD.

**Fig. 5:**
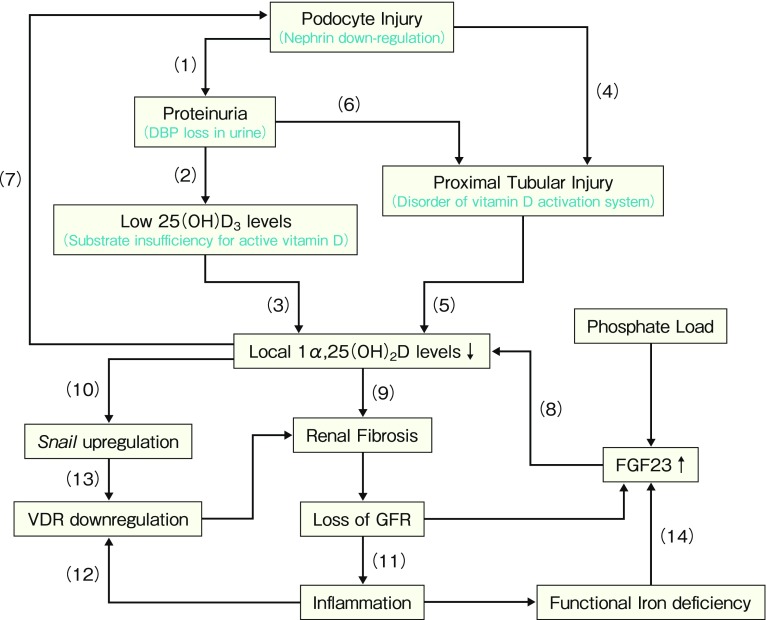
Involvement of impaired vitamin D system in the course of deterioration of renal function. This figure shows a vicious cycle of renal failure, viewed from vitamin D system. Each arrow is described in the text

In cultured proximal tubular epithelial HK-2 cells, proinflammatory tumor necrosis factor (TNF)-α inhibits the expression of VDR in a dose- and time-dependent manner [Arrow (10) in Fig. 5]. This downregulation of VDR has been confirmed in the UUO model. Because calcitriol reverses the expression of VDR in vivo, calcitriol counteracts the synergistic effect of TNF-α and TGF-β1 on EMT by inhibiting β-catenin activation [36].

The blockade of EMT may explain our finding that vitamin D prescription is associated with a lower incidence of cancer in renal transplant recipients, who are very susceptible to cancer due to the use of immunosuppressants [37]. The transcription factor, Snail, is involved not only in the metastasis (migration and invasion) of cancer, but also in the generation of cancer stem cell-like properties. Because acquisition of stem cell-like properties precedes the clinical diagnosis of cancer, Snail is a key transcription factor in the development of cancer [38].

## Vicious cycle of renal failure considering the vitamin D system

Based on these substantial data, we would like to propose a vicious cycle in the progression of CKD, as depicted in the schematic view shown in Fig. 5. Podocyte injury often results in overt proteinuria [Arrow (1)] [39, 40]. The finding that massive urinary protein is a risk factor for vitamin D insufficiency is compatible with our animal study in the PAN-nephrosis rat [15]. In this animal model, vitamin D-binding protein (DBP) is lost in urine, based on the fact that the weight of DBP is smaller than that of albumin. Because more than 99% of serum vitamin D metabolites bind DBP, DBP loss in urine causes serum 25D reduction [Arrow (2)] [41]. The positive correlation between 25D and serum calcitriol levels irrespective of CKD stage in our study [42] suggests that this 25D reduction contributes to low calcitriol levels [Arrow (3)]. Podocyte loss and the resulting adhesion between bare glomerular basement membranes and Bowman’s capsule directly induce renal tubular damage [Arrow (4)] [43]. Moreover, we previously reported that via oxidative stress, proteinuria itself damages proximal tubular epithelial cells [Arrow (6)] [44], where 25D is converted to calcitriol by 1alpha-hydroxylase. This oxidative stress also reduces the activity of 1alpha-hydroxylase [45]. Disruption in the vitamin D activation system due to renal tubular damage and high FGF23 levels results in a local shortage of its active metabolite [Arrows (5) and (8)], because FGF23 inhibits renal 1alpha-hydroxylase and stimulates 24-hydroxylase activity.

Local shortage of 1,25D in the kidney probably contributes to the development of renal fibrosis [30, 35], partly through the (partial) EMT of proximal tubules driven by the transcription factor Snail [Arrow (10)]. The milieu of inflammation often accompanies the resulting low GFR (CKD) [Arrow (11)]. Inflammatory cytokines such as TNF-α in the kidney lead to downregulation of VDR [Arrow (12)] [36], and the transcription factor Snail represses VDR expression [46] [Arrow (13)]. Hence, vitamin D signaling is further disrupted, resulting in the vicious cycle of impaired vitamin D signaling that occurs in the setting of CKD. We believe that this vicious cycle plays a role in the pathogenesis of deterioration of renal function.

## Which is renoprotective, native D or active D?

Previous basic studies including ours have documented that active vitamin D therapy attenuates kidney injury by protecting podocytes [15] and by suppressing renin transcription [29], mesangial proliferation, and interstitial fibrosis [34]. In fact, in the clinical setting, calcitriol reduces proteinuria in IgA nephropathy [47], and paricalcitol also decreases proteinuria in patients with diabetic nephropathy receiving an angiotensin converting enzyme inhibitor or angiotensin-receptor blocker [28]. A systematic review has confirmed these antiproteinuric effects [27]. However, our study showed no association between endogenous circulating 1,25D levels and CKD progression [6]. One reason may be that VDR activation by low levels of endogenous 1,25D should be much lower in CKD patients than that driven by a pharmacological dosage of an active VDR activator.

Regarding native vitamin D, a recent small uncontrolled observational study showed that oral cholecalciferol decreases albuminuria and urinary TGF-β in patients with type 2 diabetic nephropathy on renin–angiotensin–aldosterone system inhibition [48]. In this study, serum 1,25D levels increased as well as 25D levels, suggesting that the benefit of vitamin D replacement stems from an increase in 1,25D. Notably, we reported recently that administration of massive amounts of 25D exacerbates renal fibrosis in the UUO kidney in 1alpha-hydroxylase knockout mice, which cannot convert 25D to 1,25D [49]. Therefore, 25D, per se, is unlikely to protect the kidney without conversion to 1,25D.

In the OVIDS-CKD study, we also reported that high FGF23 levels predict a worse renal outcome [6]. Because FGF23 inhibits 1alpha-hydroxylase in proximal tubules and monocytes [Arrow (8) in Fig. 5] and because we failed to show an association between circulating (“systemic”) 1,25D and renal outcome, “local” 1,25D but not 25D appears to protect the kidney [Arrow (9) in Fig. 5].

In this context, the observation that 25D levels are not associated with the eGFR slope in transplant recipients with transplantation vintage greater than 10 years is reasonable (Fig. 4, right panel). This is probably because 1alpha-hydroxylase activity must be weak in recipients receiving a high dosage of a calcineurin inhibitor for a long time who had resulting proximal tubular atrophy due to striped renal fibrosis.

## The rationale for suggesting renal outcome as the fourth outcome of CKD-MBD

The following findings support the rationale for suggesting renal outcome as the fourth outcome of CKD-MBD: (1) the observed association of FGF23 and vitamin D status with renal outcome [6]; (2) administration of native vitamin D and active vitamin D reduces albuminuria and proteinuria, respectively; (3) phosphate load exacerbates renal fibrosis in an animal study [50]; (4) VDR activators attenuate renal fibrosis in an animal model; and (5) high levels of serum phosphate or FGF23 attenuate the renoprotective effect of angiotensin receptor blockade [51]. However, from the clinical point of view, what is lacking is clinical evidence showing the benefit of a phosphate binder and/or active vitamin D therapy in terms of attenuating the progression of CKD. Randomized controlled trials are needed to examine the effect of these therapies on renal outcome such as eGFR halving or initiation of RRT, before truly regarding ESRD as the fourth outcome of CKD-MBD.
